# Physicians’ Perspectives on Causes of Health Care Errors and Preventive Strategies: A Study in a Developing Country

**Published:** 2018-05

**Authors:** Abbas SHEIKHTAHERI, Monireh SADEQI-JABALI, Zahra HASHEMI-DEHAGHI

**Affiliations:** 1. Health Management and Economics Research Center, Iran University of Medical Sciences, Tehran, Iran; 2. Dept. of Health Information Management, School of Health Management and Information Sciences, Iran University of Medical Sciences, Tehran, Iran; 3. Dept. of Health Information Technology, Faculty of Paramedical, Kashan University of Medical Sciences, Kashan, Iran; 4. Dept. of Management and Accounting, School of Management, Islamic Azad University, South Tehran Branch, Tehran, Iran; 5. Eye Research Center, Tehran University of Medical Sciences, Tehran, Iran

**Keywords:** Medical error, Healthcare error, Patient safety, Physician

## Abstract

**Background:**

To prevent health care errors, the main causes and preventive strategies should be identified. The purpose of this study was to identify the causes and preventive strategies of health care errors from the perspectives of physicians.

**Methods:**

We surveyed 250 randomly selected physicians in five teaching hospitals in Tehran, Iran, in 2015. We used a questionnaire with 29 questions regarding causes and 17 ones regarding the preventive strategies. The participants were asked to answer the questions based on Likert’s five-point score (1=very low to 5= very high). The data was analyzed using descriptive (frequency, and mean scores) and inferential statistics in SPSS.

**Results:**

Managerial factors (3.6±0.7), personal factors of providers (3.5±0.6), factors related to the patients (3.4±0.71), and the factors pertinent to laboratory and pharmacy (3.2±0.8) were the main causes respectively. The most important preventive strategies were improvement of academic education, better taking past medical history, implementing electronic prescription and increasing healthcare budget.

**Conclusion:**

Heavy workloads, long work shifts, failure to do thorough examination and to collect detailed history information, providers’ fatigue, patients’ reluctance to follow orders or to give their complete information, failure to give detailed instruction to patients about the medications, lack or insufficient monitoring and supervising systems, and lack of enough budget were some of the most important causes of errors. Using IT to access patients’ information, improving patients’ adherence, reducing workload, developing efficient methods for collecting patients’ information, dedicating adequate budget for improvement programs are recommended.

## Introduction

Patient safety is one of the key aspects of quality of healthcare systems. Medical errors and risks to patient safety have always threatened the patients’ health (1). American Institute of Medicine defines medical errors and unwanted events as negligence in observing the programmed measures and/or following a wrong method to reach a specific purpose (2,3). Errors might take place during diagnosis, giving prescription, surgery, employing medical equipment, preparing lab reports and so on (4). Negative outcomes such as increase of mortality rate, infections, variety of disabilities, physical injuries, and considerable costs incurred to compensate the patients are some the consequences of health care errors (1).

Death rate due to medical errors in the USA is more than that by breast cancer, car accidents, or HIV (5). In Germany, 100000 medical errors led to 25000 deaths per year (2). Medical errors in Australia are responsible for 18000 preventable deaths and more than 50000 patients are disabled each year by medical errors (6). Results of a review study on medical errors reported a rate of 52 medical errors per 100 admissions and 24 medical errors per 1000 patient days (7).

Along with development and expansion of health services in Iran, we observe increase in number of medical errors; although there is no accurate report in this regard (8). About 3%–17% of the hospitalized patients have suffered unwanted side effects caused by medical errors and around 30%–70% of these were preventable (9).

Likewise other professions, errors in medicine are not completely avoidable; still, it is possible to design a system to minimize the probability and negative effects of the errors (6). The global approach in health systems is toward recognizing the structures leading to attenuation of medical errors as possible (10). The first approach to decrease medical errors is to detect the causes of the errors (11). In addition, preventing and detecting the causes of medical errors is imperative (12). Following preventive strategies by healthcare organizations is highly effective in cutting the number of medical errors (13).

Medical errors in American health system are due to improper communications, physicians’ errors in prescribing drugs and problems in health information systems. In addition, factors such as large population and inefficient and poor communication among clinical wards are effective in increase of the rate of medical errors (14). Under-staffed nurses and medical team members, workload, fatigue, and work pressure on medical team members were of the most noted causes of medical errors by about half of the physicians (15). Poor education provided to health staff, workload, stressful work condition and increase of patient to nurse ratio were some of the reported causes of medicine-related errors (16).

There is paucity of data on medical errors and the ways of preventing them in Iran, and in light of this, the present study was an attempt to determine causes of and strategies of preventing medical errors in public hospitals in Tehran, Iran.

## Materials and Methods

The study was carried out as a cross-sectional survey in 2015. The study population was comprised of physicians working in educational hospitals affiliated with Tehran and Iran Universities of Medical Sciences (including general practitioners, residents, and specialists). At first, five hospitals were selected randomly and sample size – based on Morgan’s table- was obtained 300. Sample size for each hospital was determined relative to the number of physicians working in that hospital and the participants from each hospital were selected randomly.

A questionnaire was used for data gathering. The questionnaire consisted of demographical questions, questions on the participants’ viewpoint regarding the causes of medical errors (n=29), and questions on the participants’ attitudes regarding errors prevention strategies (n = 17). The questions were designed based on literature review. The questions were designed based on Likert’s five-point score (very low, low, moderate, high, very high). In addition, we included an open-ended question and the participants were asked to add other causes and methods they found necessary to be taken into account.

To ascertain validity of the questionnaire, it was provided to three experts in medical errors and patient safety fields and modifications were made in the questionnaire based on their opinions. As to reliability, Cronbach’s alpha was employed and the alpha for the causes of errors and preventing solutions were obtained 0.92 and 0.88 respectively. In addition, the questionnaire was filled out by 10 physicians (pretest) two times with 10 d intervals and the results showed a reliability of 84%.

The questionnaires were distributed among the participants who filled the questionnaire in presence of the researcher. If more time was needed by the physicians, the questionnaires would be collected later. Eventually, 250 out of 300 questionnaires were returned. The responses were scored (1=very low to 5=very high). Afterward, the data were analyzed by descriptive statistics (frequency, and mean scores) and inferential statistics (t-test, ANOVA, and non-parametric tests in the case of non-normal distribution of data) in SPSS16.0 (Chicago, IL, USA).

To classify the causes and the strategies, factor analysis method with Varimax rotation was employed. According to factor analysis, the questions pertinent to the causes were categorized in four categories of factors related to healthcare providers (n=11), laboratory and pharmacy factors (n=6), patient-related factors (n=7), and organizational/managerial factors (n=5). We categorized the questions of preventive strategies into following categories: health care providers (n=2), education (n=3), information technology (n=2), and organizational/managerial strategies (n=10).

### Ethical observations

Prior arrangements were made with the managers of the hospitals and the wards’ directors and all codes and regulations were observed. In addition, the participants were volunteers and expressed their willingness verbally. Confidentiality of the information was observed throughout the study.

## Results

Male participants constituted 55.3% of the study participants, 45.9% were residents, 29.8% were general practitioners, and 15% were specialists. Average age of the physicians was 39.5±6.1 and average work experience was 8.6±4.3.

Among four factors, the participants mentioned managerial factors (3.6±0.7) as the main factor effective on medical errors followed by personal factors of the providers (3.5±0.6), factors related to the patients (3.4±0.71), and the factors pertinent to laboratory and pharmacy (3.2±0.8).

Table 1 lists the viewpoints of the physicians regarding the causes of medical errors.

**Table 1:** Viewpoints of the physicians regarding the causes of medical errors

***Variable***		***Very high and high***	***Moderately***	***Low and very low***	***Mean ± SD***
Factors pertinent to the providers of health care services	Unreadability of the physicians’ orders	127 (49.8)	86 (33.7)	38 (14.9)	3.43±1.05
	Physicians or nurses’ negligence	113 (44.3)	110 (43.1)	28 (11)	3.41±0.95
	Wrong diagnosis	115 (45.1)	96 (37.6)	38 (14.9)	3.42±0.98
	Failure to take the medical record into account	121(47.5)	98 (38.4)	34 (13.3)	3.43±0.94
	Inaccurate examination	173 (67.8)	56 (22)	18 (7)	3.83±0.97
	Lack of enough knowledge, experience and skill in diagnosing and treatment	129 (50.7)	96 (37.6)	28 (11)	3.49±0.94
	Fatigue	145 (56.7)	92 (36.1)	14 (5.5)	3.81±0.92
	Long and frequent work shifts	175 (68.6)	66 (25.9)	6 (2.4)	4.02±0.84
	Unsuitable physical/mental conditions when the error occurs	125 (49)	94 (36.9)	34 (13.3)	3.52±0.94
	Familiarity with and skill of working with equipment	85(33.3)	118(46.3)	48(18.8)	3.14±0.92
	Failure to observe instructions and protocols	94(36.9)	111(43.5)	44(17.3)	3.22±0.95
Factors pertinent to the patients	Failure to give an accurate explanation of the symptoms and history	117(69.4)	64(25.1)	10(3.9)	3.84±0.78
	Failure to mention the medicines already used	140(54.9)	91(35.7)	22(8.7)	3.61±0.89
	Patient’s failure to follow medication instruction	155(60.8)	74(29)	24(9.4)	3.76±0.98
	Faking disease	86(33.7)	117(45.9)	50(19.6)	3.23±0.1
	Refuse to mention addictions due to legal concerns	116 (45.5)	86 (33.7)	51 (20)	3.35±0.1
	Failure to mention special habits or behaviors due to social concerns	86 (33.7)	125 (49)	40 (15.6)	3.29±0.97
	Communicational problems	74 (29)	110 (43.1)	67 (26.3)	3.08±0.98
Factors pertinent to laboratory and pharmacy	Samples taken from wrong person	93 (36.5)	80 (31.4)	74 (29)	3.13±1.23
	Inaccuracy in labeling the samples	106 (41.6)	83 (32.5)	62 (24.4)	3.20±1.07
	Contamination of the sample during sampling and testing	108 (42.4)	96 (37.6)	47 (18.5)	3.37±1.01
	Wrong explanation about using/not using medicine	119 (46.7)	100 (39.2)	32 (12.6)	3.44±0.92
	Similarity of medicine names	87 (34.1)	114 (44.7)	50 (19.6)	3.20±0.95
	Wrong packaging	60 (23.5)	107 (42)	84 (32.9)	2.8±1.06
Managerial factors	Lack of enough budget for medical services	177 (69.4)	64 (25.1)	12 (4.7)	3.99±0.89
	Failure to control and monitoring	141 (55.3)	82 (32.2)	30 (11.8)	3.63±0.92
	Lack of education program for the personnel	147 (57.7)	84 (32.9)	18 (7.1)	3.69±0.85
	Problems in instructions/policies/standards	126 (49.4)	95 (37.3)	32 (12.6)	3.51±0.92
	Problems with information systems (e.g. unavailability of patient’s information when needed)	124 (48.6)	91 (35.7)	38 (14.9)	3.44±0.95

The most important factors related to providers were long work shifts, inaccurate examination, and fatigues of the health care providers. Among the factors pertinent to the patients, the participants mentioned failure to note the exact symptoms and history, failure to indicate the exact drugs used by the patient, and failure to follow physicians’ orders as the most important causes. As to the causes pertinent to laboratory and pharmacy, contamination of the samples and the staff’s failure to inform patients regarding the exact medicine consumption schedule were the main causes of the errors. In addition, the results indicated no significant differences regarding the mentioned causes of medical errors based on age, gender, education, and work experience of the participants.

Physicians’ viewpoint regarding the main approaches to reduce the rate of errors showed that they believed strategies related to providers (4.1±0.64) were the most effective approaches. The next most effective approaches, in a descending order, were educational approaches (3.9±0.67), managerial approaches (3.8±0.71), and information technology approaches (3.7±0.81). Improvement of academic education process, more accurate examination of the patients, rewarding staff with lower error rate, increase of budget and more financial support for health services, during work shifts and work hours, were the most important solutions to reduce the rate of errors from the physicians’ viewpoint (Table 2). There was no significant difference between proposed solutions based on age, gender, education, and work experience of the participants. The participants mentioned other causes such as unauthorized physicians graduated in other countries who work in clinics without license, employing unqualified personnel in pharmacies, physicians’ failure to examine patient thoroughly, wrong diagnosis, excessive para-clinical measures due to lack of time to examine the patient records, and lack of proper supervision on the personnel and the physicians.

**Table 2:** Physicians’ viewpoint regarding strategies to prevent medical errors

***Variable***		***Very high and high***	***Moderately***	***Low and very low***	***Mean ± SD***
Education	Improvement of academic education process	211(82.8)	30(11.8)	12 (4.7)	4.2± 0.88
	Consulting and asking for help	194 (76.1)	47 (18.4)	12 (4.7)	3.9 ± 0.78
	Mandatory educations on the systems and the equipment	148 (58)	93 (36.5)	12 (4.7)	3.7 ± 0.84
IT	Employing electronic systems (e.g. electronic prescription)	139 (54.5)	100 (39.2)	12 (4.7)	3.7 ± 0.88
	Employing computerized decision-support system (e.g. alarm system)	133 (52.1)	102 (40)	16 (6.3)	3.7 ± 0.87
Providers	Accuracy in taking patient’s history and examination	215 (84.3)	26 (10.2)	4 (1.6)	4.2 ± 0.72
	Reviewing the patient’s records, and family history	195 (76.4)	50 (19.6)	6 (2.4)	3.9 ± 0.72
Management	Providing decent work environment (lighting, temperature, ventilation, etc.)	185 (72.5)	52 (20.4)	12 (4.7)	3.98 ± 0.86
	Increase health budget for medical services	211 (82.7)	36 (14.1)	2 (0.8)	4.3 ± 0.75
	Rewarding staff with lower error rate	187 (73.3)	54 (21.2)	10 (3.9)	4.06 ± 0.88
	Motivating personnel to study and update their knowledge	167 (65.5)	70 (27.5)	8 (3.2)	3.9 ± 0.84
	Routine control and monitoring	179 (70.2)	64 (25.1)	8 (3.1)	3.87 ± 0.75
	Codifying clear regulations, by-laws, and standard protocols	159 (62.3)	66 (25.9)	22 (8.6)	3.75 ± 0.89
	Holding routine in-service training courses	144 (56.5)	95 (37.3)	12 (4.7)	3.7 ± 0.87
	Motivation evidence based medicine	152 (59.6)	87 (34.1)	10 (3.9)	3.8 ± 0.86
	Designing disciplinary codes for negligence of regulations	106 (41.6)	99 (38.8)	48 (18.8)	3.3 ± 1.03
	Reducing work hours and congested work shifts	207 (81.2)	40 (15.7)	2 (0.8)	4.2 ± 0.73

In addition, they emphasized on developing safety reporting system and analyzing patient safety data for detecting incidents and causes.

## Discussion

We investigated the Iranian physician’s point of view regarding the most important causes and prevention strategies of health care errors and found that they considered factors related to management, healthcare providers, patients and laboratory/pharmacy, respectively, as the most important causes.

Among the factors pertinent to the providers, long work shifts were of the main causes of the errors. Heavy workload contributed to medicine-related errors, which is consistent with other studies (17–20). Workload and the providers’ stress was the main causes of medicine-related errors (16). Similar studies were reported the same (21–23). Blendon et al. reported that lack of enough nurses (53%), workload, pressure, and weariness of the providers (50%) were the main causes of errors by the half of the physicians in the study (15). Other studies reported similar results (24,25). Our participants emphasized on reducing work shift hours and congestion of work shifts to prevent errors. The majority of the physicians believed that development of medical errors prevention systems (55%), and increase in number of nurses (51%) were two effective approaches in reducing medical errors (15). Creating a reasonable relationship between the number of personnel and patients in different clinical wards, and reducing workloads and work hours may prevent fatigue in the personnel, improve nurses’ concentration, and prevent human errors (26). In general, one may conclude that the issue of workload is a common problem in many countries’ health systems, which needs supplying adequate workforce and solving the problem of long work shifts.

Fatigue of the providers, consistent with other studies (22, 25, 27) was one of the common causes of healthcare errors. About 3%–10% of medical errors resulted from fatigue of providers (28). Frequency of medical errors was higher among the residents who expressed more fatigue (29). There was significant relationship between mental weariness and severity of medical errors (30). Scheduling a proper work condition is an influential risk management strategy in many industries including healthcare (31). Reduction of workload must be one of priority of the managers.

Failure to examine the patient thoroughly was another cause of medical errors. In Khuzestan, Iran, physicians in emergency wards did not possess the required knowledge to determine patients’ condition and triage and taking medical history (32). One of the main requirements of providers was to receive education on how to examine the patients and taking history as many of the physicians were not good in this regard (33). Failure to taking complete medical history was one of the main causes of medical errors (34). The physician is required to conduct all diagnostic measures including asking about the symptoms, doing physical examinations, using clinical and para-clinical tests, and consulting others if needed. Therefore, to avoid medical errors, any medical intervention must be done after complete examinations and taking history. It is essential to provide adequate education on the effect of taking medical history as prevention strategies of medical errors as indicated by the many of our participants.

Negligence of the physician’s orders by the patient was one of the main causes of errors pertinent to the patients. The patient’s active participation was highly effective in preserving their health and avoiding medical errors (35–37). Proper relationship between the physician and the patient and development of a proper emotional atmosphere between the patient and the physician led to higher adherence to the physician’s orders by the patients and better medical results (38). In addition, providing enough information to patients about their conditions can result in better cooperation between patients and the health providers (36); while, failure to share the information with patients leads to negative consequences for patients. Patients who are not aware of their situations are more probable to neglect physicians’ order (39). However, many patients are deprived of information about their conditions (36,37). In this respect, using information technologies such as email may improve participation of patients in treatment (40). Mobile applications reduce risk of forgetting therapeutic instructions and improve adherence to the instructions (41). Additionally, access to electronic health records improves physician’s access to patient’s information. Observing confidentiality codes also makes the patient more eager to share their information and reduces the risks consequently.

The results indicated inappropriate educational programs as one of the causes of errors. Poor knowledge about the prescribed drugs was one of the main causes of medical errors in the eastern Mediterranean countries (42). In Saudi Arabia, the lack of experience and pertinent education was one of the main causes of medical errors as well (43). Similar results were indicated the same (16). Moreover, education level and work experience of the nurses were related to the errors pertinent to medicines (44). Thereby, improvement of educational process and holding re-education courses for the graduates are imperative. The participants considered improvement of academic education as one the most important strategies. Managerial issues were among the causes of errors. Issues such as lack of adequate budget and lack of educational programs or supervision were highlighted by the physicians. Lack of adequate resources is one of the challenges that rural hospitals are faced with for ensuring safety of patients and quality of health care services (45). Several factors are effective on errors, and managers need to follow different strategies based on the causes (46). In this regard, following the standards, and providing appropriate work environment are recommended.

In addition, participants stated that developing error reporting systems and analysis of patient safety data are necessary. Reporting systems are needed to gather and analysis of patient safety incident data (3, 47). The causes of errors can be determined and proper interventions can be taken by using information about the errors. In this regard, using administrative databases are useful for patient safety analyses; however, quality of these databases should be improved (48).

This study was limited to a few hospitals. In addition, only self-reporting by the physicians was taken into account. Generalization of the results to all hospitals may not be possible. In addition, the causes of errors and solutions can be widely variable and there must be factors that are not included in this study. Therefore, there is a need for further studies in this field.

## Conclusion

Physicians considered factors related to managerial and organizational issues (such as insufficient budget, lack of monitoring and inappropriate education) and factors related to providers (long working shifts, inappropriate examination and taking history as well as fatigue) as the main causes of healthcare errors. They also found measures such as improvement of professional behavior (e.g. complete examination and taking history) and then educational and managerial approaches as the most important preventive strategies. In addition, all of physician, regardless of their age, sex, education, and experience have similar views regarding causes and preventive strategies of healthcare errors.

## Ethical considerations

Ethical issues (Including plagiarism, informed consent, misconduct, data fabrication and/or falsification, double publication and/or submission, redundancy, etc.) have been completely observed by the authors.
